# Vitamin B6 deficiency and diseases in elderly people – a study in nursing homes

**DOI:** 10.1186/1471-2318-13-13

**Published:** 2013-02-08

**Authors:** Ida K Kjeldby, Gunvor S Fosnes, Solveig C Ligaarden, Per G Farup

**Affiliations:** 1 Department of Research, Innlandet Hospital Trust, Brumunddal, Norway; 2 Unit for Applied Clinical Research, Department of Cancer Research and Molecular Medicine, Norwegian University of Science and Technology, Trondheim, Norway; 3 Department of Medicine, Innlandet Hospital Trust, Gjøvik, Norway

## Abstract

**Background:**

Vitamin deficiency is a cause of health related problems in elderly people. The aims were to study associations between vitamin B6 (B6) and diseases (primarily functional gastrointestinal disorders) in elderly people in nursing homes, the prevalence of B6 deficiency and factors associated with B6 deficiency.

**Methods:**

This cross-sectional study included residents in nursing homes. Demographics, nutritional status (Mini Nutritional Assessment, MNA® ), physical activity, activity of daily living (Katz Index), dietary habits, use of drugs, and psychiatric and somatic diseases were recorded. A blood sample was collected for haematological and biochemical screening, including B6 (p-PLP); p-PLP values < 20 nmol/l indicates B6 deficiency. The results are given as mean (SD).

**Results:**

Sixty-one residents (men/women: 22/39) with an age of 85.3 (6.8) years and BMI 25.7 (4.5) kg/m^2^ were included. Malnutrition and risk of malnutrition were present in 11.5% and 61% respectively. Dietary intake of B6 (mg/day) in men and women were 1.60 (0.30) and 1.18 (0.31) (recommended 1.6 and 1.2 respectively), and 14 (23%) used B6 supplements. Median p-PLP was 20.7 (range <4.0-175.8), 30 subjects (49%) had B6 deficiency. B6 deficiency was associated with old age, low s-alanine aminotransferase and s-albumin, elevated s-homocysteine and inactivity (p-values 0.01-0.03). There were no clinically significant associations between B6 deficiency and somatic or psychiatric disorders, and B6 deficiency was not observed in subjects given B6 supplements.

**Conclusions:**

Half of the residents had vitamin B6 deficiency. Vitamin supplement was effective prophylaxis for deficiency and should be recommended to all elderly people in nursing homes.

## Background

Multiple diseases and complaints, nutritional problems and the natural aging process contribute to the high prevalence of health related problems in elderly people. In nursing homes, the prevalence of malnutrition is in the order of 14 – 71%. Malnutrition, diseases and complaints, of which gastrointestinal (GI) disorders are among the most common ones, are associated with high costs and reduced quality of life [1-6].

Vitamin B6 (B6) is water-soluble and consists of the vitamers pyridoxine, pyridoxamine, pyridoxal, and their respective 5’-phosphateesters. Pyridoxal 5’-phosphate (PLP) is the biologically active form. B6 is a coenzyme in various metabolic pathways including degrading of homocysteine (Hcy). It exerts a wide range of functions in the human body and has been associated with cancer, cardiovascular events, seizures, migraine, chronic pain, depression, cognitive failure, immune deficiency etc. [7-12]. We have previously reported an association between low intake of B6 and symptom load in subjects with Irritable Bowel Syndrome (IBS) [13]. B6 interferes with the serotonin metabolism and is a P2X receptor antagonist. Both serotonin and the P2X receptor in the enteric nervous system are related to the GI function. The interference with serotonin metabolism and the P2X receptor could explain the association between B6 intake and symptoms in subjects with IBS [1,14].

The prevalence of B6 deficiency in European institutionalized elderly people varies from < 1 – 75% [15-17]. The official Norwegian recommendations for daily intake of B6 for men and women above 30 years of age are 1.6 mg and 1.2 mg respectively. Studies indicate that the requirements of B6 in elderly people are higher due to decreased absorption, increased catabolism and impaired phosphorylation [18,19]. Elderly people are at risk of B6 deficiency because they consume less food, in particular food containing B6.

The aims were to study associations between vitamin B6 deficiency and diseases, primarily functional gastrointestinal disorders (FGID) because of our previous findings of an association between B6 deficiency and abdominal complaints, the prevalence of B6 deficiency and factors associated with B6 deficiency in elderly people in nursing homes [13].

## Methods

### Study design and study population

This cross-sectional survey was part of a larger study in nursing homes in two Norwegian counties (Hedmark and Oppland) in 2009 [20]. Registered and auxiliary nurses with the best knowledge of the residents collected data from the medical records and from interviews with the residents. In subjects unable to give reliable information due to cognitive impairment, supplementary information was given by their next of kin. The nursing staff filled in the data in questionnaires. A blood sample was drawn, immediately light protected, centrifuged, and transported in a cooler until kept in a freezer at minus 70 degree C.

Residents above 60 years of age with a stay in the nursing home for more than 8 weeks were included. Excluded were those with organic gastrointestinal diseases or a planned discharge within two weeks.

### Variables

Demographics, smoking habits, use of alcohol, all intakes of drugs (classified according to the ATC classification system) and their use (regular or on demand) and number of drugs (polypharmacy was defined as regular use of more than three drugs) were registered, as was type of nursing home department (regular unit/ dementia unit/ rehabilitation unit/ psychogeriatric unit/ other). Nutritional status was assessed with Mini Nutritional Assessment (MNA®, total score 0–30, where <17 indicates poor nutritional state, 17–23.5 risk of poor nutritional state, and >23.5 not at risk) [21]. Diet was registered as type of food (tube nutrition/ mashed food and soups/ bread without crust/ ordinary food) and number of meals per day. A simplified diet registration was completed for one week with calculation of intake of fluids (amount and type), dietary fibre and B6. Physical ability was registered as physical activity (steps/day) and activities of daily living (ADL) (Katz Index of Independence in ADL [22], score 0–6, where high score indicates independence). Present and previous somatic and psychiatric diseases and degree of present complaints were registered with special focus on GI symptoms. A blood sample was analyzed for pyridoxal 5-phosphate (p-PLP, reference value >20 nmol/L, values < 20 nmol/l indicates B6 deficiency [23]; the limit was confirmed by the laboratory at St. Olavs Hospital which performed the analyses), homocysteine (Hcy, <15 μmol/L), albumin (age 40–69: 36–45 g/L, age ≥70: 34–45 g/L), haemoglobin, haematocrit, sodium, potassium, calcium, bilirubin, alanine aminotransferase (ALAT, females: 10–45 U/L, males: 10–70 U/L), alkaline phosphatase, thyroid stimulating hormone and creatinine. Glomerular filtration rate (GFR) was calculated with Cockcroft-Gault formula: normal >90 mL/min; severe reduction (kidney failure) < 15 mL/min.

Daily dietary intake of B6 was calculated from the diet registration, and the content of B6 in the diet was based on the Norwegian Food composition table [24].

Constipation and diarrhoea were defined according to the Rome III criteria for functional constipation and diarrhoea [25], but since cognitive impairment made information about insufficient criteria for IBS unreliable, subjects with IBS-constipation and IBS-diarrhoea were included in the groups with constipation and diarrhoea respectively, as were subjects with regular use of laxatives and loperamide. The degree of abdominal pain/ discomfort was calculated as the product of frequency (graded 0–4; 0 = 0–1 days per week, and 4 = 6–7 days per week) and severity (graded 0–3; none; mild = hardly affects the residents; moderate = affects the residents, but does not impair ADL; and severe = impairs ADL), total score 0–12. Stool frequency, consistency (according to the Bristol Stool Form Scale, score 1–7)*,* straining, incomplete evacuation, sensation of ano-rectal obstruction and manual manoeuvres to facilitate defecation were registered for assessment of bowel function and degree of constipation and diarrhoea. Table 1 shows the details. Total scores were calculated by adding the scores of each variable. Low scores indicate normal function.

**Table 1:** Gastrointestinal complaints with scoring

**Variables**	**Grading**	**Bowel function**	**Constipation**	**Diarrhoea**
Stool frequency	≤1 stool per week	2	3	0
	1-2 stools per week	1	2	0
	3-6 stools per week	0	1	0
	1 stool daily	0	0	0
	2-3 stools daily	0	0	1
	4-5 stools daily	1	0	2
	≥6 stools daily	2	0	3
Stool consistency: “Bristol Stool Form Scale”	1. Separate hard lumps, like nuts	2	2	0
	2. Sausage-shaped but lumpy	1	1	0
	3. Like a sausage or snake but with cracks on its surface	0	0	0
	4. Like a sausage or snake, smooth and soft	0	0	0
	5. Soft blobs with clear-cut edges	0	0	0
	6. Fluffy pieces with ragged edges, a mushy stool	1	0	1
	7. Watery, no solid pieces	2	0	2
Straining	≥25% of defecations		1	
	< 25% of defecations		0	
Manual manoeuvres	≥25% of defecations		1	
	< 25% of defecations		0	
Feeling of incomplete evacuation	≥ 25% of defecations		1	
	< 25% of defecations		0	
Sensation of ano-rectal obstruction	≥ 25% of defecations		1	
	< 25% of defecations		0	
Sum score (range)		0 – 4	0 – 9	0 – 5

The numbers are the scores related to each symptom with the sum score at the bottom. Low scores indicate normal function.

### Statistics

Student t-test and Mann–Whitney U-test were used for comparisons of continuous variables with and without normal distribution, the exact unconditional z-pooled test for comparisons of proportions [26], Mann–Whitney U-test and linear-by-linear association test for comparisons of ordinal outcomes, and Spearman’s rho for correlation analyses. P-values ≤ 0.05 were considered statistically significant. The analyses were performed with SPSS version18, StatXact version 9, and http://www.stat.ncsu.edu/exact/.

To show a correlation of r = − 0.30 between p-PLP and abdominal pain/discomfort, 85 participants were required (given α= 0.05 and 1-β = 0.80).

### Ethics

The study was approved by the Regional Committee for Medical Research Ethics in Trondheim and The Norwegian Data Inspectorate represented by the Privacy Ombudsman for Research at Oslo University Hospital, and performed according to the Declaration of Helsinki. Informed consent was given by the resident or by their next of kin if the resident was unable to consent.

## Results

### Participants’ characteristics

**Figure 1:**
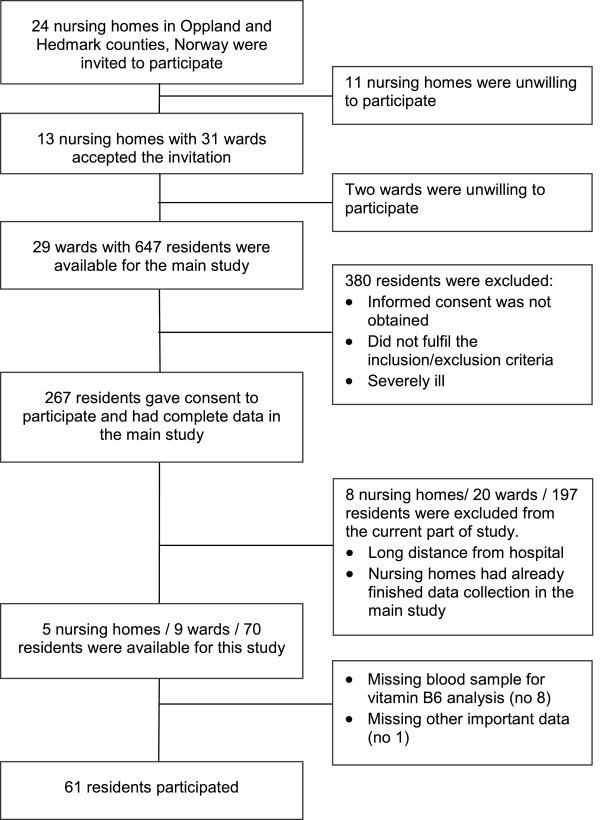
Flow chart of the participants in the study.

Out 24 nursing homes invited to participate, 13 accepted the invitation and in these nursing homes 267 nursing home residents participated in the main study and 61 of them (39 [64%] women and 22 [35%] men) were included in this study. Figure 1 shows the selection of subjects. The mean age and BMI of the participants were 85.3 years (range 66 – 99 years) and 25.7 kg/m^2^ (range 14.3 – 36.2 kg/m^2^) respectively. Thirty-seven (60.7%) resided in regular nursing home units and 24 in specialized units for demented. In all 48 participants (78.7%) had dementia, 20 (32.8%) had suffered from a stroke, and 28 (45.9%) had present or previous hypertension or were treated with anti hypertensives. Malnutrition (MNA-score < 17) and risk of malnutrition (MNA-score 17–23.5) were present in 6/52 (11.5%) and 32/52 (61.5%) participants respectively. Median p-PLP was 20.7 nmol/L, 30 participants (49.2%) had plasma levels indicating B6 deficiency. Tables 2 and 3 give the participants’ characteristics, and complaints and diseases.

**Table 2:** The participants’ characteristics and comparisons between participants with and without B6 deficiency

**Variable**	**All participants n=61**	**Participants with p-PLP deficiency n=30**	**Participants with normal p-PLP n=31**	**p-value**
Females	39 (63.9%)	20 (66.7%)	19 (61.3%)	0.667
Age (years)	85.3 (6.8)	87.2 (6.1)	83.5 (7.0)	0.032
Able to give informed consent	12 (19.7%)	5 (16.7%)	7 (22.6%)	0.607
Reside in dementia units	24 (39%)	10 (33%)	14 (45%)	0.351
Body mass index (kg/m^2^) (n=54)	25.7 (4.5)	25.2 (4.7)	26.1 (4.3)	0.467
p-PLP (nmol/L)	20.7 (<4.0-175.8)			
Dietary vit. B_6_ (mg/day) females (n=38)	1.18 (0.31)	1.13 (0.28)	1.22 (0.34)	0.353
Dietary vit. B_6_ (mg/day), males (n=22)	1.60 (0.30)	1.53 (0.35)	1.66 (0.26)	0.332
MNA-score (n=52)	22.5 (11–27)	21.5 (11–27)	22.5 (13.5-26.5)	0.172
Dietary grain products (items/day)	11.6 (3.7)	11.6 (4.6)	11.6 (2.8)	0.984
Dietary fibre (g/day)	18.4 (6.6)	18.6 (8.1)	18.2 (4.8)	0.791
Steps per day (n=54)	200 (0–5000)	100 (0–1500)	500 (0–5000)	0.016
Katz’ ADL-index^1^ (n=60)	4 (0–6)	4 (0–6)	3 (0–6)	0.127
ALAT below reference value (< 10 U/L)	14 (23.0%)	11 (36.7%)	3 (9.7%)	0.014
Albumin (g/L)	39.1 (3.6)	38.0 (3.9)	40.1 (2.9)	0.022
Homocysteine (μmol/L)	18.6 (10.5)	21.5 (11.6)	15.8 (8.5)	0.032
Glomerular filtration rate^2^ (mL/min) (n=56)	56.6 (19.9)	55.4 (22.2)	57.7 (18.0)	0.660
Number of drugs used	6 (1–13)	5 (1–13)	6 (1–13)	0.372
Polypharmacy (> 3 drugs)	49 (80.3%)	21 (70.0%)	28 (90.3%)	0.052
Use of vit. B_6_ supplements (ATC A11)	14 (23.0%)	0 (0.0%)	14 (45.2%)	<0.001
Use of sedatives/ hypnotics (ATC N05C)	14 (23.0%)	2 (6.7%)	12 (38.7%)	0.003
Use of SSRI (ATC N06AB)	23 (37.7%)	7 (23.3%)	16 (51.6%)	0.023
Use of anti-dementia drugs (ATC N06D)	16 (26.2%)	4 (13.3%)	12 (38.7%)	0.025

^1^ Score 0–6. Low score indicates independence in ADL.

^2^ Estimated by the Cockroft-Gault formula.

The results are given as no. (percent), mean (SD), and median (range). p-values indicate the difference between the groups with and without B6 deficiency. n=61 if not specified.

**Table 3:** The participants’ diseases and comparisons between participants with and without B6 deficiency

**Variable**	**All participants n=61**	**Participants with p-PLP deficiency n=30**	**Participants with normal p-PLP n=31**	**p-value**
Gastrointestinal pain/ discomfort^1^	0 (0–6)	0 (0–4)	0 (0–6)	0.288
Bowel function^1^	0 (0–3)	0 (0–2)	0 (0–3)	0.167
Constipation^1^ (n=59)	2 (0–5)	2 (0–4)	2 (0–5)	0.336
Diarrhoea^1^	0 (0–2)	0 (0–2)	0 (0–2)	1.000
Constipation	33 (54.1%)	17 (56.7%)	16 (51.6%)	0.689
Diarrhoea	6 (9.8%)	4 (13.3%)	2 (6.5%)	0.529
Other abdominal diseases^2^	15 (24.6%)	5 (16.7%)	10 (32.3%)	0.198
Cancer	8 (13.1%)	3 (10.0%)	5 (16.1%)	0.530
Cardiovascular diseases^3^	49 (80.3%)	25 (83.3%)	24 (77.4%)	0.607
Cerebrovascular disease	20 (32.8%)	11 (36.7%)	9 (29.0%)	0.565
Hypertension (needing treatment)	28 (45.9%)	18 (60.0%)	10 (32.3%)	0.040
Venous thromboembolism	4 (6.6%)	3 (10.0%)	1 (3.2%)	0.310
Haematological diseases^4^	4 (6.6%)	2 (6.7%)	2 (6.5%)	1.000
Dementia	48 (78.7%)	24 (80.0%)	24 (77.4%)	0.852
Parkinson’s disease	2 (3.3%)	0 (0.0%)	2 (6.5%)	0.206
Cognitively impaired^5^	59 (96.7%)	30 (100%)	29 (93.5%)	0.206
Psychiatric diseases^6^	31 (50.8%)	15 (50.0%)	16 (51.6%)	0.929
Musculoskeletal diseases^7^	23 (37.7%)	12 (40.0%)	11 (35.5%)	0.796
Pulmonary diseases^8^	12 (19.7%)	5 (16.7%)	7 (22.6%)	0.607
Diabetes	5 (8.2%)	2 (6.7%)	3 (9.7%)	0.762
Thyroid disease	7 (11.5%)	3 (10.0%)	4 (12.9%)	0.803
Allergy	4 (6.6%)	3 (10.0%)	1 (3.2%)	0.310

^1^ The variables for gastrointestinal pain/ discomfort, bowel function, constipation and diarrhoea were graded 0–12, 0–4, 0–9, and 0–5 respectively.

^2^ Biliary or hepatic disease, previous abdominal surgery, other abdominal disease.

^3^ Heart disease, peripheral circulatory dysfunction, hypertension, stroke.

^4^ Anaemia, leukaemia.

^5^ Unable to give informed consent, dementia, use of anti-dementia drugs.

^6^ Psychosis, schizophrenia, neurosis, depression, anxiety.

^7^ Osteoporosis, Bechterew’s disease, arthritis, osteoarthritis, fibromyalgia.

^8^ Recurrent pulmonary infections, asthma, chronic obstructive pulmonary disease.

The results are given as median (range) or number (percent). p-values indicate the difference between the groups with and without B6 deficiency. n=61 if not specified.

### Vitamin B6 deficiency

Table 2 gives the characteristics of the subjects with and without B6 deficiency, and comparisons between the groups. Subjects with B6 deficiency were statistically significantly older, were less physically active (steps per day), had lower levels of s-ALAT and s-albumin, higher levels of homocysteine and used less sedatives/hypnotics, selective serotonin reuptake inhibitors (SSRI) and anti-dementia drugs. Normal p-PLP values were seen in all the 14 subjects using vitamin supplements containing B6, in contrast to 17 out of 47 (36%) subjects not using vitamin supplements. Other characteristics, such as sex, BMI, and dietary intake of B6, fibre and grain products showed no significant differences between the groups. MNA-scores were correlated with p-PLP levels (rho=0.27; p=0.05) and intake of grain products with intake of B6 (rho=0.56; p<0.001).

### Vitamin B6 deficiency and diseases

The morbidity was high, 54 residents (88.5%) had 3 or more diseases and complaints. Except for hypertension, no significant associations were seen between B6 deficiency and diseases (Table 3). Neither were GI complaints, which were primarily focused on, associated with B6 deficiency. The correlations (Spearman’s rho) between p-PLP and abdominal pain/ discomfort, bowel function, constipation and diarrhoea were 0.04 (95% CI: -0.21;0.29), p=0.75; 0.09 (95% CI: -0.19:0.37), p=0.48; 0.07 (95% CI: -0.21:0.35), p=0.58; and 0.00 (95% CI: -0.27:0.27), p=0.99 respectively.

## Discussion

The finding that half of the residents had B6 deficiency defined as p-PLP < 20 nmol/L was discouraging. The cut-off level was in accordance with the literature, and the local laboratory has shown that the 2.5 percentile in a healthy adult population with their method was 23 nmol/L with a decline with age (personal communication) [19,23,27]. High prevalence rates of B6 deficiency (51-75%) has also been reported in other studies in elderly people in institutions and hospitals [16,17]. On the other hand, Gonzalez-Gross et al. reported p-PLP < 20 nmol/L in only one out of 218 Spanish institutionalised elderly people [15]. B6 deficiency has been shown to be common also in the adult population with prevalence rates of 11-24% [19,27]. In all, B6 deficiency seems to be a common problem in elderly people in nursing homes and occurs also in subjects without malnutrition. No symptoms and signs are specific for B6 deficiency. In this study, as in other reports, malnutrition, inactivity, old age, low s-albumin and s-ALAT values, and high homocysteine values were associated with B6 deficiency and seem reasonable to have in mind [8,15,17,28,29].

Mean dietary intake of B6 was in large in accordance with Norwegian recommendations, but the prevalence of B6 deficiency was nevertheless high. Also in subjects with B6 deficiency the intake was only marginally below the recommendations. This supports the assumption that the need of B6 is increased in elderly people, and indicates that the recommendations need adjustment for age [18,19]. Vitamin supplements were not included in the calculation of dietary intake of B6 and could explain the lack of significant associations between dietary intake of B6 and p-PLP. An important finding was that vitamin supplements efficiently protected against B6 deficiency. None of the users of vitamin supplements had B6 deficiency. Recommendation of vitamin supplement to all elderly people in institutions is an easy and safe prophylaxis.

The nutritional state was unsatisfactory, malnutrition was present in 11.5% and only 27% were at no risk of malnutrition. Even more unsatisfactory findings have been reported in other studies in nursing homes with malnutrition prevalence as high as 71% [5]. Malnutrition should be unnecessary and has been associated with high costs [1]. Poor nutritional status, old age, inactivity and low s-albumin were all related to the overall poor health of subjects with B6 deficiency.

The study excluded a strong and clinically significant association between B6 and FGID in this group of elderly people. One explanation of the discrepancy between this study and our findings in a previous study in younger patients with IBS is that the pathogenesis and pathophysiology of FGID in elderly people differs from that in younger subjects with IBS [13]. An exact diagnosis of IBS is based on precise information about the symptom’s duration and relation to defecation, which was impossible to obtain in this group. The diagnosis of IBS was therefore omitted in this study and replaced by the unspecific diagnosis of FGID. P2X receptor antagonists like PLP attenuate gut sensitivity after infections [14]. This might be a cause of symptoms in subjects with IBS, but perhaps not in elderly people with FGID. Nor were associations with other common disorders such as dementia, cognitive impairment, psychiatric disorders and cardiovascular diseases shown. The discrepancy between our findings and the reported associations between B6 deficiency and somatic and psychiatric diseases in other studies could be due to a high degree of morbidity in this study in elderly people or a type II error. The association between B6 and hypertension was judged as clinically insignificant and might have occurred by chance. The lack of associations between B6 deficiency and diseases/disorders in this study is no excuse for not preventing B6 deficiency in elderly people. For unknown reasons, subjects with normal/ high p-PLP used more sedatives/ hypnotics, SSRI, and anti-dementia drugs*.*

### Strengths and limitations

The population characteristics, such as gender, age, BMI and nutritional status, and the prevalence of dementia, cardiovascular diseases etc. were, with some variation, comparable with most other studies in nursing homes in Norway and other countries [2-5,15]. Compared with our main study, participants in this study might have had somewhat better nutritional status and been less dependent on support in ADL. Data from non-participants in the main study were not available, but a biased selection seems unlikely. Exceptionally poor health status did not explain the high prevalence of B6 deficiency.

Data quality might have been variable. Demographics and background variables were classified according to a national classification system, drugs according to ATC classification, and nutrition and activity of daily living with validated questionnaires (MNA and Katz). These data probably have high quality. The nursing staff collected data from the participants, their medical records and their next of kin. The high proportion of participants with cognitive impairment and next of kin’s often insufficient knowledge might have reduced the data quality. The nursing staff’s knowledge of the residents was, however, good, since all participants had stayed in the nursing home for more than eight weeks. Because resources in the nursing homes are limited, exact registration of intake of food was difficult and might have been inadequate and explains in part the poor correlation between intake of B6 and p-PLP.

When planning the study, a clinically significant correlation between p-PLP and GI symptoms was considered to be below minus 0.3. Although the number of subjects in the study was smaller than planned, the study excluded a clinically significant association between B6 deficiency and GI symptoms. The lower limit of any of the 95% CI of the correlations between p-PLP and GI symptoms was minus 0.27.

Multivariable analyses were performed but turned out to be unreliable. The limited number of subjects in the study and the high number of variables made a reduction in the number of variables necessary, and the results varied depending on the method used for reduction of the variables. Multivariable analyses were therefore omitted. The low number of subjects might have influenced the conclusions and the generalisability of the results.

## Conclusions

Half of the residents in nursing homes had B6 deficiency and malnutrition was common. Old age, inactivity, low s-albumin and s-ALAT levels, and high s-homocysteine levels were associated with B6 deficiency. Poor nutritional status was associated with low B6 values, but B6 deficiency occurred also in subjects without malnutrition. In this study, no associations of clinical significance were seen between B6 deficiency and diseases, but such associations are well known from other reports. Vitamin supplements prevented B6 deficiency in all subjects and should be recommended to all elderly people in nursing homes.

## Competing interests

The authors declare that they have no competing interests.

## Authors’ contributions

IKK has written the protocol for this add-on project, applied for approval by the authorities, collected parts of the data, prepared the clean data file, analysed the data and written the paper. GSF has been responsible for the main project, from which this add-on project was planned, has collected most of the data and registered and classified all use of drugs, and participated in the analyses and preparation of the paper. SCL has been responsible for the registration of the diet and calculation of the content of the diet, and has participated in the preparation of the paper. PGF is the guarantor and has been responsible for the integrity of the work as a whole, from inception to published article. All authors have read and approved the last version of the paper.

## Pre-publication history

The pre-publication history for this paper can be accessed here:

http://www.biomedcentral.com/1471-2318/13/13/prepub
